# The Role of Grass Compost and *Zea Mays* in Alleviating Toxic Effects of Tetracycline on the Soil Bacteria Community

**DOI:** 10.3390/ijerph19127357

**Published:** 2022-06-15

**Authors:** Jadwiga Wyszkowska, Agata Borowik, Jan Kucharski

**Affiliations:** Department of Soil Science and Microbiology, University of Warmia and Mazury in Olsztyn, 10-727 Olsztyn, Poland; agata.borowik@uwm.edu.pl (A.B.); jan.kucharski@uwm.edu.pl (J.K.)

**Keywords:** antibiotics in soils, microbial community, soil degradation, soil oxidoreductases, compost fertility

## Abstract

Given their common use for disease treatment in humans, and particularly in animals, antibiotics pose an exceptionally serious threat to the soil environment. This study aimed to determine the response of soil bacteria and oxidoreductases to a tetracycline (Tc) contamination, and to establish the usability of grass compost (G) and *Zea mays* (Zm) in mitigating adverse Tc effects on selected microbial properties of the soil. The scope of microbiological analyses included determinations of bacteria with the conventional culture method and new-generation sequencing method (NGS). Activities of soil dehydrogenases and catalase were determined as well. Tc was found to reduce counts of organotrophic bacteria and actinobacteria in the soils as well as the activity of soil oxidoreductases. Soil fertilization with grass compost (G) and *Zea mays* (Zm) cultivation was found to alleviate the adverse effects of tetracycline on the mentioned group of bacteria and activity of oxidoreductases. The metagenomic analysis demonstrated that the bacteria belonging to *Acidiobacteria* and *Proteobacteria* phyla were found to prevail in the soil samples. The study results recommend soil fertilization with G and Zm cultivation as successful measures in the bioremediation of tetracycline-contaminated soils and indicate the usability of the so-called core bacteria in the bioaugmentation of such soils.

## 1. Introduction

Tetracyclines (Tc) represent a group of antibiotics with a broad spectrum of activities [1,2]. The first antibiotics from the class of tetracyclines (chlorotetracycline and oxytetracycline) were discovered at the end of 1940 s, with ultimately over 20 tetracyclines in use today [3]. They can be divided into three categories: I generation tetracyclines (chlorotetracycline, oxytetracycline, tetracycline, demeclocycline), II generation tetracyclines (doxycycline, minocycline, lymecycline, meclocycline, metacycline, rolitetracycline), and III generation ones (tigecycline, omadacycline, sarecycline) [2,4]. The I generation antibiotics are naturally synthesized, whereas the II and III generation ones are semi-synthetic.

Considering data derived from 103 countries, the World Organization for Animal Health [5] has reported tetracyclines to be the most frequently used antimicrobial agents for animals worldwide. In 2017, they accounted for 33.9% of all administered drugs, on average, followed by polypeptides (11.1%), penicillins (10.9%), and macrolides (10.4%). A slightly different situation is observed in the European countries [6]. Based on data derived from 31 European countries (30 UE/UOG Member States and Switzerland), the European Medicines Agency has demonstrated tetracyclines (30.4%), followed by penicillins (26.9%), and sulfonamides (9.2%) to be the greatest contributors to the total sales of antimicrobial agents in 2017. In 2020, the demand for tetracyclines changed, making them second (26.7%) after penicillins (31.1%) and followed by sulfonamides (9.9%) in the total sales of these agents. This was the first year when the sales of penicillins surpassed those of tetracyclines. Noteworthy is that the World Health Organization classifies tetracyclines as antimicrobial agents of great importance to human medicine [7].

Their beneficial antimicrobial properties and low production costs compared to other antibiotics have made them widely applicable not only in human [8,9] and animal therapies [10] but also in agriculture [3,11]. The antibiotics from the class of tetracyclines deployed most often for therapeutic purposes in animal production include chlorotetracycline, oxytetracycline, and tetracycline [3]. In many countries, tetracyclines are also often added to feedstuffs for livestock and poultry, and are used in aquaculture as growth stimulants [10,12,13,14]. Antibiotics are poorly metabolized under in vivo conditions [1,15]. According to Daghrir and Drogui [16], 70% of the tetracycline group antibiotics are excreted with human and animal urine and excreta and released in the active form to the natural environment. The continuous release of Tc residues to the environment [17,18] and the fact that low temperature contributes to the retardation and even complete inhibition of their degradation in the soil [19] make them ubiquitous in the soil environment (Table 1).

Tetracyclines usually pervade the soil environment after the use of fertilizers contaminated with these antibiotics [17,26]. Types of veterinary drug residues in soil are associated with animal species being manure providers. The greatest amounts of antibiotics are found in the manure derived from poultry and livestock production, while lesser ones in the cow’s manure [23,24,25,26,27,28,29]. Antibiotics pervade the arable soils also as a result of field irrigation with wastewater [30,31,32]. The highest detection rate of antibiotics in the world, reaching almost 100% in soils and 98% in surface water, has been reported in China [27]. A far better situation is observed in the European countries [33]. According to Conde-Cid et al. [23], antibiotics can be detected in approximately 40% of manures and 17% of soils therein. In addition, the highly hydrophilic character and low volatility of antibiotics make them highly stable in surface and ground waters [16,34,35], and even in drinking water [36].

Given the above, they pose severe environmental problems, including ecological hazards and adverse effects on human health. These problems additionally lead to the emergence of antibiotic resistance genes in the environment [34,37]. Considering high amounts of tetracyclines used worldwide, their monitoring, including identifying responses of soil microorganisms and determining enzymatic activity, seems to be of key importance. Today, little is known about the basic changes in the community of microorganisms involved in the accelerated degradation of tetracyclines in the soil. Therefore, the aims of this research were to: (1) determine the response of soil bacteria and oxidoreductases to tetracycline (Tc), (2) establish the effect of *Zea mays* (Zm) cultivation on bacteria and oxidoreductases in the soil contaminated with Tc, and (3) identify the usability of compost from various grass species (G) in restoring the microbiological homeostasis of tetracycline contaminated soil. The gathered information may facilitate the choice of bacteria most active in tetracycline degradation and an effective bioremediation method.

## 2. Materials and Methods

### 2.1. Study Objective

The soil (Eutric Cambisol) to be analyzed was sampled from the topsoil layer (0–20 cm) of an arable field located in North-Eastern Poland (53.7167° N, 20.4167° E), where organic fertilizers have not been administered five years prior to soil sampling. According to fraction size classification by the International Union of Soil Sciences and the United States Department of Agriculture [38], the soil was classified as sandy loam (sand—0.0–2.0 mm—63.61%, dust 0.02–0.05 mm—32.68%, and loam <0.002 mm—3.71%). Compost used in the study derived from aerobic composting of cut and pre-dried grass. Table 2 presents the basic properties of the soil and compost used in the study.

Tetracycline (C_22_H_24_N_2_O_8_) was purchased at Sigma-Aldrich (Saint Louis, MO, USA). Its molecular weight is 444.4 g moL^−1^. It is highly soluble in water (231 mg dm^−3^) and features a low octanol-water partition coefficient (Log K_OW_), i.e., −1.30, pointing to its hydrophilic nature. A tetracycline (Tc) molecule has acidic functional groups, namely: tricarbonyl methane (pKa~3.3) and phenolic diketone (pKa~7.8) [23,51,52,53,54].

### 2.2. Experiment Design

A pot experiment was carried out in a greenhouse of the University of Warmia and Mazury in Olsztyn (Poland) in 2021, in polyethylene pots having the following sizes: 15 cm (height), 14 cm (pot bottom diameter), and 16 cm (pot upper diameter). Each pot was filled with 3.5 kg of sandy loam sieved through a screen with a mesh diameter of 5 mm. The experiment was established in a randomized block design and was conducted in five replications for the control soil and the soil with tetracycline addition. The following experimental variants were tested: (1) non-sown soil (C); (2) non-sown soil with the addition of tetracycline (CTc); (3) non-sown soil fertilized with grass compost (CG); (4) non-sown soil with the addition of tetracycline fertilized with grass compost (CTcG); (5) soil sown with maize (Zm); (6) soil with tetracycline and sown with maize (ZmTc); (7) soil sown with maize and fertilized with grass compost (ZmG); and (8) soil sown with maize, contaminated with tetracycline, and fertilized with grass compost (ZmTcG). The same mineral fertilization was applied in all soil variants, including both the non-sown and sown ones. Fertilizer doses were established considering nutritional demands of maize (*Zea mays* L.) of LG 32.58 variety (variety registered in the European Union), which served as the experimental crop. Maize was selected for this study because it is one of the two crops most often cultivated on all continents [55], and also because its acreage and yield continuously increase, expecting to grow by 4% and 10% until 2030, respectively, compared to 2020 [56]. The following fertilization was applied in the experiment, in mg kg^−1^ DM soil: N—140, P—50, K—140, and Mg—20. Nitrogen was used in the form of urea, phosphorus as potassium dihydrophosphate, potassium as potassium dihydrophosphate and potassium chloride, and magnesium as magnesium sulfate heptahydrate. Tetracycline (Tc) was applied in a dose of 100 mg kg^−1^ DM soil in respective experimental variants (CTc; TcG; ZmTc; ZmTcG). This Tc dose reflects adverse scenarios involving high doses of antibiotics pervading the soil as a result of their uncontrolled disposal to municipal sewage or their storage in landfills [57,58]. Compost from grasses (G) was used in the amount of 4 g C kg^−1^ DM soil to ensure natural biostimulation of the soil microbiota in respective experimental variants (CG; CTcG; ZmG; ZmTcG).

Mineral fertilizers, tetracycline, and compost were applied to the soil once before it had been placed in pots. Afterward, the soil was hydrated using distilled water to 60% of the water capillary capacity, and soil from selected experimental variants (Zm; ZmTc; ZmG; ZmTcG) was sown with *Zea mays* L. (Zm) in the amount of 6 seeds. After the emergence (at the BBCH 10 stage), maize plants were thinned and only 4 were left in each pot. The experiment lasted 50 days, and soil moisture content was kept stable throughout this period at 60% of the water capillary capacity. Daytime ranged from 15 h 5 min to 17 h 5 min, the average temperature was at 14.9 °C, and the average air humidity was at 67.5%. The plants were grown under natural light. The leaf greenness index (SPAD, Soil and Plant Analysis Development) was determined twice (on day 25 and day 50 just before the plants had been cut). Determinations were carried out in 8 replications, using a SPAD 502 Chlorophyll Meter 2900P. At the BBCH 51 stage (beginning of panicle emergence), analyses were conducted to determine the yield of aerial parts and roots of maize. The plants were dried at a temperature of 60 °C for 4 days.

### 2.3. Methods of Laboratory Analyses

The count of microorganisms and the activity of enzymes were determined in fresh soil, moist soil, and soil sieved through a screen with 2 mm mesh diameter, whereas the physicochemical properties were tested in dry soil.

#### 2.3.1. Microbiological Analyses

- Count of microorganisms

On days 25 and 50 of the experiment, the numbers of soil microorganism colonies (cfu) were determined with the serial dilution method. In order to extract a microbial community, a soil suspension was prepared (10 g of dry soil bulk in 90 cm^3^ of a sterile 0.85% NaCl solution) and shaken for 30 min. Afterward, a series of dilutions were prepared and used to isolate organotrophic bacteria [46], oligotrophic and copiotrophic bacteria [47], and actinobacteria [48]. Culture conditions and detailed procedure of microorganism isolation were described in our previous work [59]. The colony numbers (cfu) of all groups of microorganisms were determined in 4 replications.

- DNA isolation

DNA was isolated from the soil using a Genomic Mini AX Bacteria+” kit (A&A Biotechnology, Gdansk, Poland). DNA isolation was followed by enzymatic lysis conducted using Lyticase (cat. 1018-10, A&A Biotechnology, Gdansk, Poland) and mechanical lysis performed on a FastPrep—24 type apparatus using zirconia beads. The resulting bacterial DNA was additionally purified by means of an Anti-Inhibitor Kit (A&A Biotechnology, Gdansk, Poland). The presence of bacterial DNA in the tested samples was confirmed in the Real-Time PCR performed in a CFX Connect thermocycler (Biorad), using a SYBR Green dye as fluorochrome and universal primers 1055F (5′-ATGGCTGTCGTCAGCT-3′) and 1392R (5′-ACGGGCGGTGTGTAC-3′) [60].

- Amplicon sequencing

Amplicons of the taxonomic groups Bacteria and Archea were sequenced based on the hypervariable V3-V4 region of the 16S rRNA gene. In the case of bacteria, amplification was performed using specific primer sequences 341F (5′-CCTACGGGNGGCWGCAG-3′) and 785R (5′-GACTACHVGGGTATCTAATCC-3′). PCR conditions were provided in detail in our previous work [61]. The sequencing was conducted at the Genomed SA company (Warsaw, Poland). DNA was sequenced on a MiSeq sequencer (Illumina, San Diego, CA, USA), in the paired-end (PE) technology using an Illumina v2 kit (San Diego, CA, USA).

- Bioinformatic analysis

The sequences obtained were subjected to quality control. Incomplete and chimeric sequences were discarded. The bioinformatic analysis ensuring the classification of bacteria to particular taxonomic levels was performed using the QIIME software package based on reference sequence database GreenGenes ver. 13_8. The results were presented as the percentage of relative abundance of sequences identified at selected taxonomic levels (phylum and genus). This manuscript presents results with OTU (operational taxonomic units) exceeding 1%. The sequencing data were deposited in the GenBank NCBI (https://www.ncbi.nlm.nih.gov/) (accessed on 27 March 2022) under accession numbers of ON042235-ON042332.

#### 2.3.2. Biochemical Analyses

As in the case of microbial count determination, i.e., on days 25 and 50 of the experiment, soil samples from each replication were determined in another 3 replications for the activity of selected enzymes from the class of oxidoreductases: Deh—dehydrogenases (EC 1.1), and Cat—catalase (EC 1.11.1.6). All determinations were carried out with standard methods presented in Table 2. The specific procedures of the enzymatic test (buffers, incubation temperature and duration, reaction arrest time) were provided in detail in our previous works [59,62]. The activity of dehydrogenases was determined using a Perkin-Elmer Lambda 25 spectrophotometer (Woburn, MA, USA) at a wavelength (λ) of 485 nm. The enzymatic activity was defined as the amount of product released by 1 kg DM soil per 1 h, And hence, Deh activity was expressed in μMol triphenylformazan, whereas Cat activity—in Mol O_2_.

#### 2.3.3. Chemical and Physicochemical Analyses

Before the experiment, the collected soil was determined for fraction size with the aerometric method and for contents of: total nitrogen (N_total_); organic carbon (C_org_); soil organic matter (SOM); available P, K, and Mg; and exchangeable cations Ca^2+^, Mg^2+^, K^+^, and Na^+^. In addition, before the experiment had been established and after plant harvest, soil pH was measured in 1 mol KCl dm^−3^ and soil samples were determined for hydrolytic acidity (HAC) and sum of exchangeable base cations (EBC). The HAC and EBC values obtained were used to compute the cation exchange capacity (CEC) and alkaline cation saturation (ACS). All determinations were made in three replications. The above-mentioned determinations were carried out with standard methods presented in Table 2 and described in our previous work [62].

### 2.4. Data and Statistical Analysis

The counts of organotrophic bacteria and actinobacteria (cfu) were used to determine the colony development index (CD) and the ecophysiological diversity index (EP) [63]. The CD and EP values were computed from Equations (1) and (2), respectively:

CD = [N1/1 + N2/2 + 3/3….. N10/10] × 100;(1)

where: N1, N2, N3,...N10—sum of the quotients of colony numbers of microorganisms identified in particular days of the study (1, 2, 3, ... 10) and the sum of all colonies in the entire study period;

EP = −Σ(pi × log10 pi)(2)

where: pi—the quotient of the number of colonies of microorganisms from particular days of the study and the sum of all colonies from the entire study period.

The effect of tetracycline on soil microorganisms and enzymes was determined based on the index of tetracycline effect (IF_Tc_) on soil microorganisms and enzymes (3):

(3) $${\mathrm{IF}}_{\mathrm{Tc}}=\frac{A_{\mathrm{Tc}}}{A}-1$$

where: A_Tc_—the number of microorganisms or the activity of enzymes in the soil with the addition of tetracycline, A—the number of microorganisms or the activity of enzymes in the control soil.

Additional analyses were conducted to evaluate the effect of maize cultivation (IF_Zm_) and soil fertilization with grass compost (IF_G_) on the colony numbers of microorganisms and activities of soil enzymes. The IF_Zm_ and IF_G_ values were computed from Equations (4) and (5), respectively:

(4) $${\mathrm{IF}}_{\mathrm{Zm}}=\frac{A_{\mathrm{Zm}}}{A}-1$$

where: A_Zm_—the number of microorganisms or the activity of enzymes in the soil sown with maize, A—the number of microorganisms or the activity of enzymes in the non-sown soil.

(5) $${\mathrm{IF}}_{G}=\frac{A_{G}}{A}-1$$

where: A_G_—the number of microorganisms or the activity of enzymes in the soil fertilized with grass compost, A—the number of microorganisms or the activity of enzymes in the non-fertilized soil.

The operational taxonomic units (OTU) of bacteria established at all taxonomic levels allowed computing the Shannon-Wiener index (H’) of bacterial diversity (6):

H’ = −Σp_i_ × (lnp_i_) (6)

where: p is the ratio of OTU number of one representative of the tested taxon to the total OTU number of the entire taxon.

All results were subjected to a statistical analysis using Statistica 13.3 package [64]. Firstly, Kruskal-Wallis and Shapiro-Wilk tests were conducted to establish the normality of the data distribution. Next, two-way analysis of variance (ANOVA) was carried out to determine the effect of tetracycline and compost on the microbiological, enzymatic, and physicochemical properties of the soil. All significant differences between mean values were determined using the post-hoc Duncan test or post-hoc Dunn test with Bonferroni correction at a 95% confidence interval.

The data from the metagenomic analysis were developed using bioinformatic software for statistical calculations and graphical visualization. The phylum of bacteria was compared statistically by means of the G-test (w/Yates’) + Fisher test, using STAMP 2.1.3 software [65]. The analyzed data was presented with the confidence interval of 95%. The collected metagenomic data related to the phylum and genus of bacteria was analyzed using the R v1.2.5033 software [66] with R v3.6.2 [67] and a gplots library [68]. The OTU ≥ 1% data was presented on heat maps with a dendrogram of their similarities, whereas unique and common genera of bacteria identified in the soils non-sown and sown with *Zea mays*, OTU ≥ 1%, were presented on a Venn diagram plotted using InteractiVenn [69].

## 3. Results

### 3.1. Response of Zea Mays to Soil Contamination with Tetracycline

In the soil not fertilized with grass compost (G), tetracycline (Tc) did not inhibit the growth and development of *Zea mays* (Zm) (Table 3), while it increased the leaf greenness intensity at the 8th leaf stage of Zm development, increasing the SPAD value by as much as 23.8%. The aerial parts of Zm produced significantly greater biomass in the soil fertilized with G than in the non-fertilized soil. In turn, Tc effect in the fertilized soil was insignificant, just as in the non-fertilized one. In this experimental variant, Tc did not affect the SPAD value. The Zm yield was positively affected by soil fertilization with G, which increased the biomass of aerial parts by 9.3% in the soil not contaminated with Tc to 10.6% in the Tc-contaminated soil.

### 3.2. Response of Bacteria to Soil Contamination with Tetracycline

The negative values of the IF_Tc_ index proved the adverse effect of Tc on the communities of organotrophic bacteria and actinobacteria (Table 4), whereas its positive value indicated the positive impact of Tc on copiotrophic bacteria (Table S1).

Definitively lower negative IF_Tc_ values were obtained in the soil fertilized using G than in the non-fertilized soil, proving that organic matter provided to the soil with compost mitigates the adverse effects of Tc on the mentioned bacterial groups. In the case of copiotrophic bacteria, higher IF_Tc_ values were usually determined in the soil not fertilized with G (0.214–0.591) than in the fertilized soil (0.070–0.529). On day 25 of the experiment, Tc elicited an adverse effect on oligotrophic bacteria, as the IF_Tc_ values reported in this analytical period ranged from −0.262 to −0.469. The adverse Tc effect was neutralized on day 50, as indicated by IF_Tc_ values ranging from −0.009 to −0.052. An explicitly positive effect on this bacterial group was observed upon the fertilization of soil sown with Zm (IF_Tc_ = 0.134—0.687).

In the non-sown soil, the fertilization with G contributed to the greatest increase in the population numbers of actinobacteria and organotrophs (Table 5), with the increase being significantly greater in the soil contaminated with Tc than in the non-contaminated soil. The population numbers of copiotrophic bacteria remained relatively stable, whereas negative IF_G_ values were noted in most variants for oligotrophic bacteria. In the case of soil sown with Zm, fertilization with compost positively affected actinobacteria, whereas it adversely influenced oligotrophic bacteria in the soil not contaminated with tetracycline. Fertilization with compost triggered greater positive changes in the actinobacteria count in the soil contaminated with Tc than non-contaminated with Tc. In this experimental variant, compost elicited a minor effect on the other groups of the analyzed bacteria. On day 50 of the experiment, the IF_G_ values ranged from −0.060 to 0.005 for organotrophic bacteria, from −0.189 to −0.031 for oligotrophic bacteria, and from 0.032 to 0.074 for copiotrophic bacteria.

Among all independent variables tested, bacterial communities were most strongly affected by Zm cultivation. The mean results achieved in the assumed analytical periods indicate that the IF_Zm_ values determined for organotrophic and copiotrophic bacteria and actinobacteria in the soil not fertilized with G and contaminated with tetracycline were generally high, and higher than in the non-contaminated soil (Table 6), reaching 2.233, 0.168, and 3.208, respectively. In the soil fertilized with G, the IF_Zm_ values were higher for copiotrophic bacteria, actinobacteria and additionally for oligotrophic bacteria under conditions of soil exposure to Tc compared to the non-exposed soil. The mean IF_Zm_ values reached 0.186, 0.969, and 0.889, respectively, for the mentioned groups of bacteria. 

**Figure 1:**
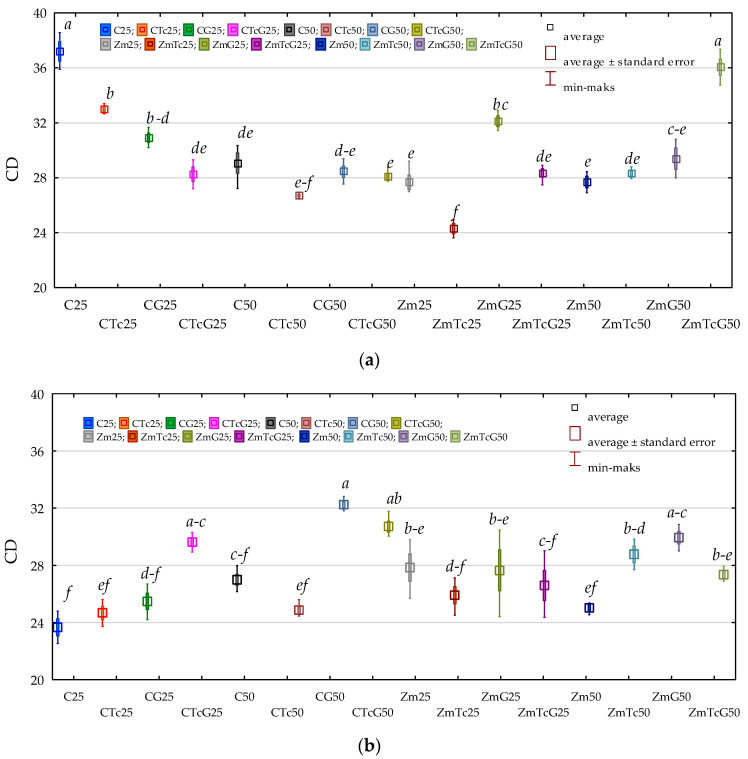
Colony development index (CD) (**a**) organotrophic bacteria (**b**) actinomycetes in soil. C—non-sown soil (control), CTc—non-sown soil contaminated with tetracycline, CG—non-sown soil fertilized with compost, CTcG—non-sown soil contaminated with tetracycline and fertilized with compost, Zm—soil sown with maize (*Zea mays*), ZmTc—soil sown with maize and contaminated with tetracycline, ZmG—soil sown with maize and fertilized with compost, and ZmTcG—soil sown with maize, contaminated with tetracycline, and fertilized with compost. 25—the date of the analysis, 25 days, 50—the date of the analysis, 50 days. Homogeneous groups denoted with letters (*a*–*f*) were calculated separately for each group of microorganisms.

The value of the colony development index (CD) determined for organotrophic bacteria was higher than that computed for actinobacteria (Figure 1). Regardless of the analytical period, the mean value of soil fertilization with G and soil contamination with Tc reached 29.720 for Org and 27.326 for Act. Soil contamination with tetracycline caused no changes in CD values determined for Org and Act. Mean differences in CD values between the soil samples non-contaminated and contaminated with tetracycline were not statistically significant despite fluctuations in certain analytical terms. Soil fertilization with G evoked an inexplicit effect on CD values calculated for the analyzed microorganisms as it caused no significant changes in CD value of Org and increased CD in Act from 25.962 to 28.689. The analysis of data collated in Figure 1 shows that CD values of Org and Act did not change significantly also upon Zm cultivation.

**Figure 2:**
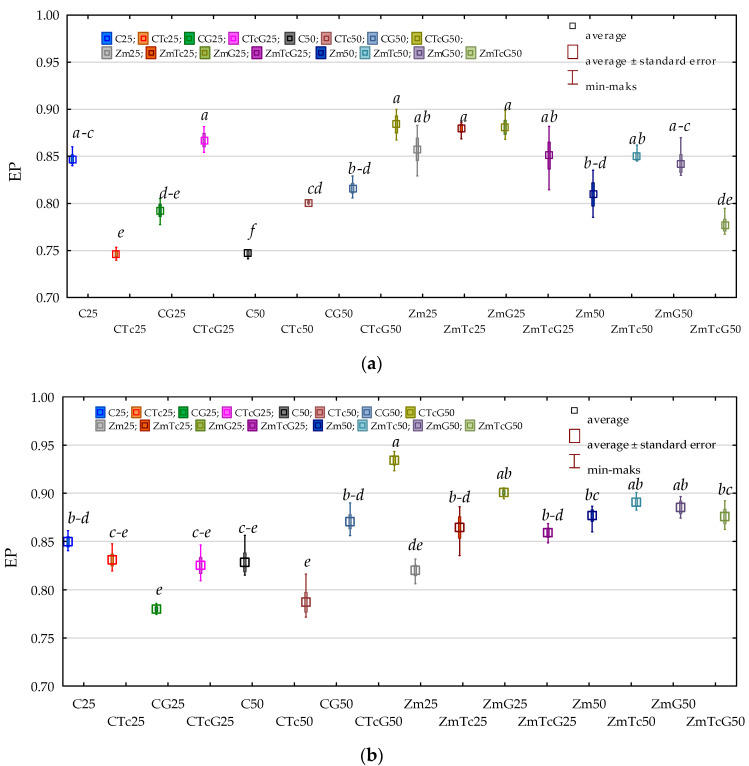
Ecophysiological diversity index (EP) (**a**) organotrophic bacteria (**b**) actinomycetes in soil. C—non-sown soil (control), CTc—non-sown soil contaminated with tetracycline, CG—non-sown soil fertilized with compost, CTcG—non-sown soil contaminated with tetracycline and fertilized with compost, Zm—soil sown with maize (*Zea mays*), ZmTc—soil sown with maize and contaminated with tetracycline, ZmG—soil sown with maize and fertilized with compost, and ZmTcG—soil sown with maize, contaminated with tetracycline, and fertilized with compost. 25—the date of the analysis, 25 days, 50—the date of the analysis, 50 days. Homogeneous groups denoted with letters (*a*–*e*) were calculated separately for each group of microorganisms.

In turn, data presented in Figure 2 indicates that the mean values of the ecophysiological diversity index (EP) computed for both Org and Act were high and reached 0.828 and 0.855, respectively. Regardless of analytical term, fertilization with G compost, and *Zea mays* cultivation, the soil contamination with Tc did not disturb the ecophysiological diversity of Org and Act. The mean EP value computed for Org was 0.824 in the non-contaminated soil and 0.832 in the Tc-contaminated soil, whereas the respective values calculated for Act were 0.851 and 0.858. Soil fertilization with G also did not disturb the ecophysiological diversity of the microorganisms tested, whereas Zm cultivation increased EP values from 0.813 to 0.844 for Org and from 0.838 to 0.872 for Act.

**Figure 3:**
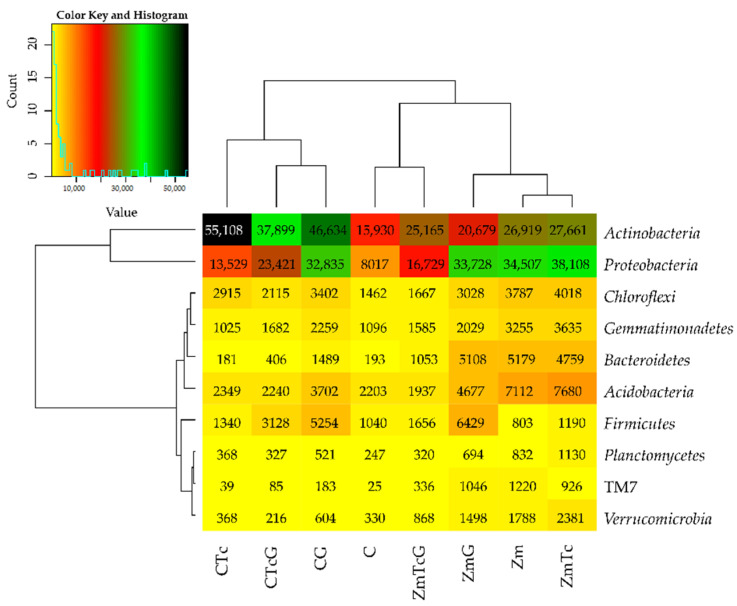
The abundance of bacterial types, OTU ≥ 1% presented by the heat map. C—non-sown soil (control), CTc—non-sown soil contaminated with tetracycline, CG—non-sown soil fertilized with compost, CTcG—non-sown soil contaminated with tetracycline and fertilized with compost, Zm—soil sown with maize (*Zea mays*), ZmTc—soil sown with maize and contaminated with tetracycline, ZmG—soil sown with maize and fertilized with compost, and ZmTcG—soil sown with maize, contaminated with tetracycline, and fertilized with compost.

The mean OTU number of the bacterial phylum reached 76,782 in the soil sown with Zm and 69,042 in the non-sown soil (Figure 3). In the non-sown soil, the value of the index of Tc effect on OTU reached 1.53, that of compost effect reached 2.17, and that of the cumulative Tc and G effect reached 1.34, whereas the respective values noted in the soil sown with Zm were at 0.07, −0.08, and −0.40. The IF value describing the effect of Zm cultivation on the soil tested reached 1.80.

**Figure 4:**
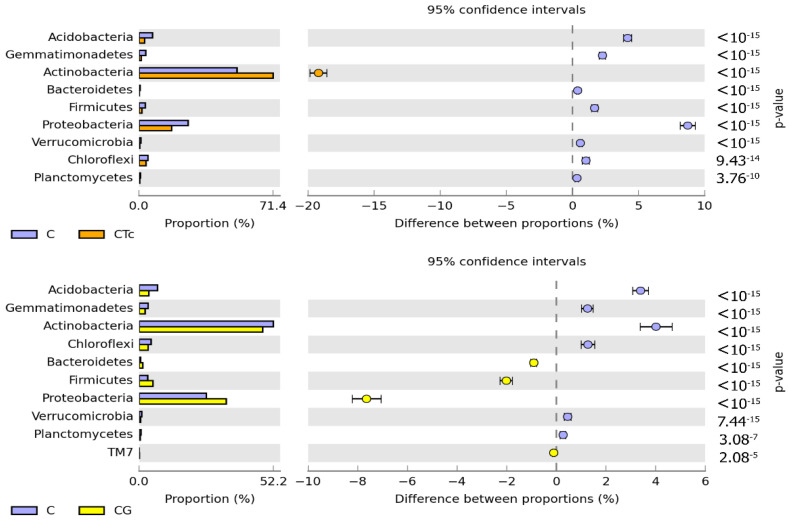

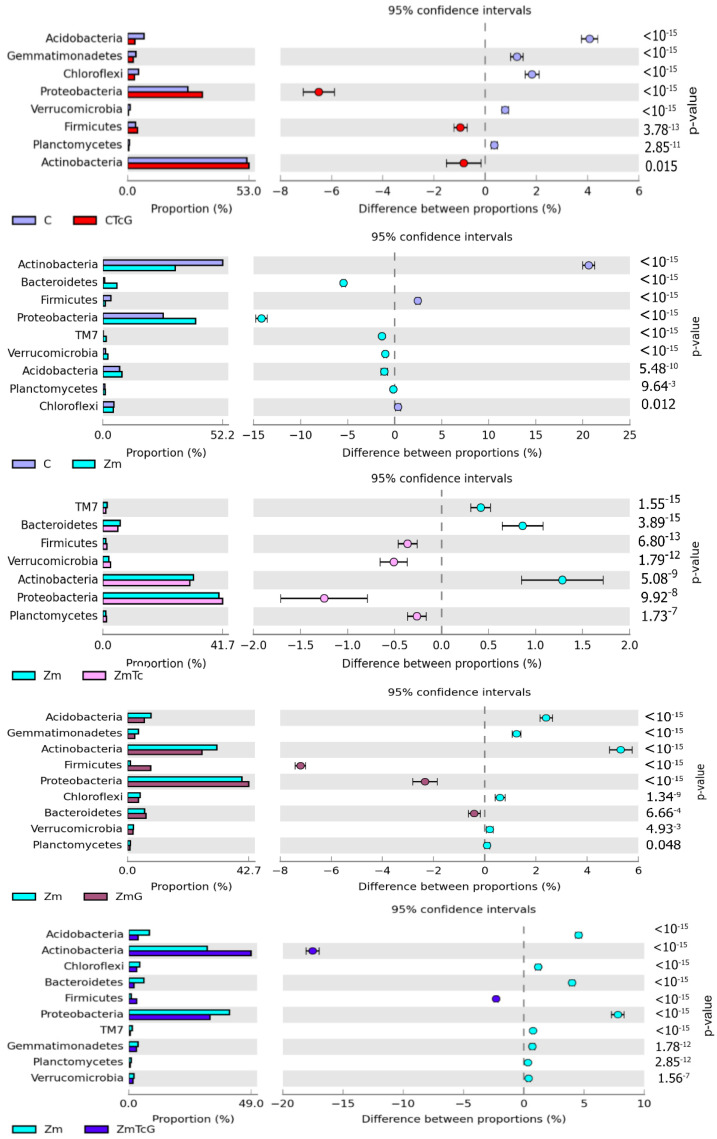
Structure of the abundance of bacterial phyla in the soil computed based on OTU ≥ 1%. C—non-sown soil (control), CTc—non-sown soil contaminated with tetracycline, CG—non-sown soil fertilized with compost, CTcG—non-sown soil contaminated with tetracycline and fertilized with compost, Zm—soil sown with maize (*Zea mays*), ZmTc—soil sown with maize and contaminated with tetracycline, ZmG—soil sown with maize and fertilized with compost, and ZmTcG—soil sown with maize, contaminated with tetracycline, and fertilized with compost.

Both in the soil sown with *Zea mays* and in the non-sown soil, the prevailing bacteria were those belonging to the *Actinobacteria* and *Proteobacteria* phyla (Figure 4).

**Figure 5:**
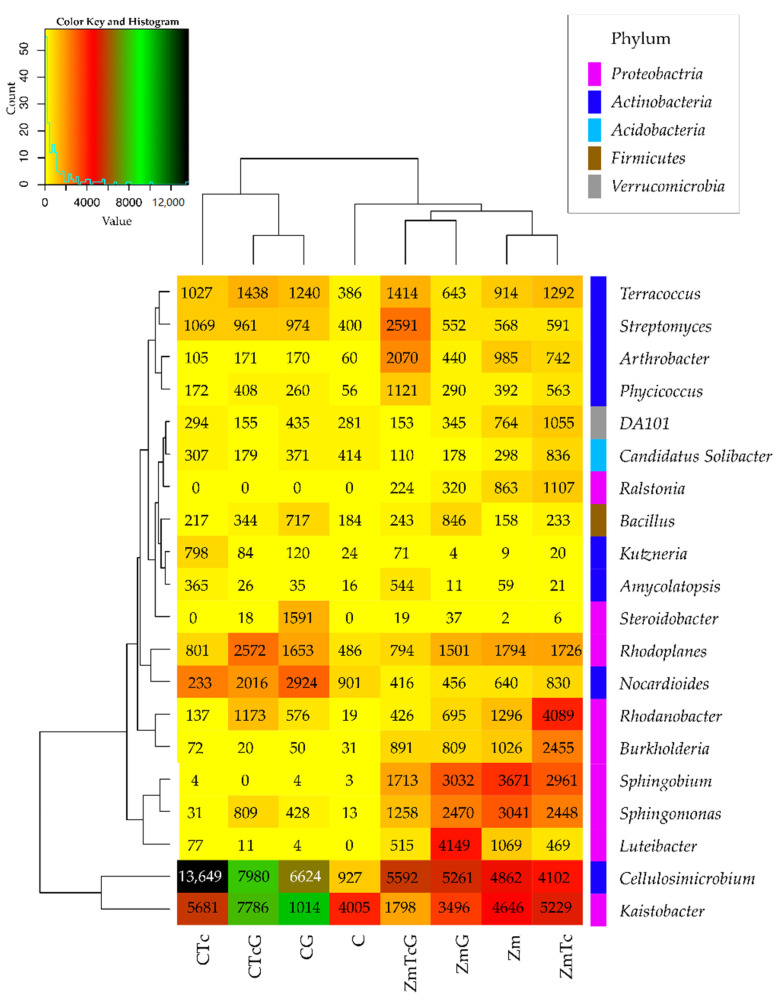
Diversity of bacterial species, presented based on OTU ≥ 1%. C—non-sown soil (control), CTc—non-sown soil contaminated with tetracycline, CG—non-sown soil fertilized with compost, CTcG—non-sown soil contaminated with tetracycline and fertilized with compost, Zm—soil sown with maize (*Zea mays*), ZmTc—soil sown with maize and contaminated with tetracycline, ZmG—soil sown with maize and fertilized with compost, and ZmTcG—soil sown with maize, contaminated with tetracycline, and fertilized with compost.

The greatest changes in the structure of both phyla were triggered by Zm cultivation, which contributed to a 20.64% decrease in the relative abundance of *Actinobacteria* and a 14.16% increase in the relative abundance of *Proteobacteria*. In this experimental variant, soil contamination with Tc decreased the relative abundance of *Actinobacteria* by barely 1.29% and increased that of *Proteobacteria* by 1.25%. The structure of the remaining bacterial phyla remained unaffected by Tc effect. In turn, soil fertilization with G diminished *Actinobacteria* contribution in the phylum structure by 5.32%, but increased that of *Proteobacteria* and *Firmicutes* by 2.33% and 7.21%, respectively. The use of this fertilizer in the Tc-contaminated soil caused a 17.52% increase in the contribution of *Actinobacteria* in the structure as well as decreased contributions of *Proteobacteria*, *Acidobacteria*, and *Bacteroides* by 7.81%, 4.55%, and 4.01%, respectively. Nine bacterial genera were identified to belong to the phylum *Proteobacteria*, eight to the phylum *Actinobacteria*, and one to each of the following phyla: *Acidobacteria*, *Firmicutes*, and *Verrucomicrobia* (Figure 5).

The prevailing genera of the phylum *Proteobacteria* included: *Kaistobacter* and *Rhodoplanes*, and these prevailing in the phylum *Actinobacteria* included: *Cellulosimicrobium*, *Nocardioides*, *Streptomyces*, and *Terracoccus*. *Kaistobacter* was the most abundant in CTc and ZmTc variants; *Cellulosimicrobium* in CTc as well as ZmTcG and ZmG; *Luteibacter* in ZmG; *Sphingomonas*, *Sphingobium*, and *Burkholderia* in the soil sown with Zm; *Rhodanobacter* in ZmTc; *Nocardioides* in CTc, CTcG, and CG; *Rhodoplanes* in CTcG; *Arthrobacter* and *Streptomyces* in ZmTcG; and *Terracoccus* in CTcG.

**Figure 6:**
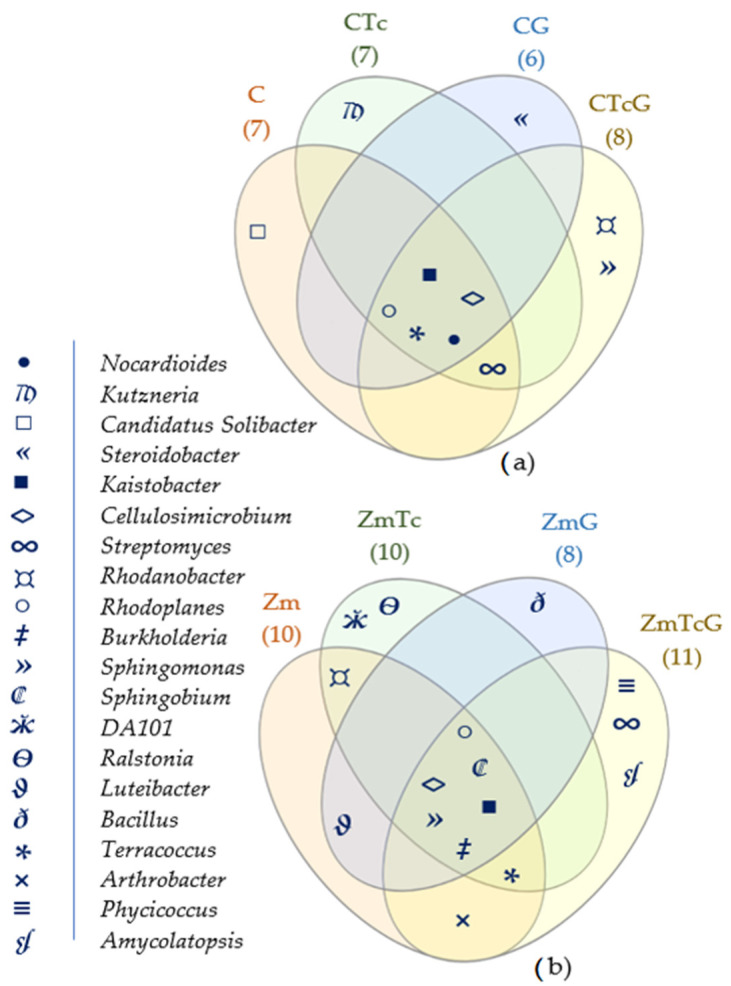
Venn diagram depicting unique and core bacterial genera in (**a**) non-sown soil and (**b**) soil sown with *Zea mays*, plotted based on OTU ≥ 1%. C—non-sown soil (control), CTc—non-sown soil contaminated with tetracycline, CG—non-sown soil fertilized with compost, CTcG—non-sown soil contaminated with tetracycline and fertilized with compost, Zm—soil sown with maize (*Zea mays*), ZmTc—soil sown with maize and contaminated with tetracycline, ZmG—soil sown with maize and fertilized with compost, and ZmTcG—soil sown with maize, contaminated with tetracycline, and fertilized with compost.

The core bacteriome identified in all variants with non-sown soil comprised the following genera: *Kaistobacter*, *Cellulosimicrobium*, *Nocardioides*, *Rhodoplanes*, and *Terracoccus* (Figure 6a). Among the aforementioned genera, only *Kaistobacter*, *Cellulosimicrobium*, and *Rhodoplanes* represented the core bacteriobiome of the soil sown with Zm (Figure 6b). These three common genera, colonizing soils regardless of their contamination with Tc, fertilization with G, Zm cultivation, and specific genera appearing exclusive in the soil contaminated with Tc (*Kutzneria*, DA101, *Ralstonia*), should be perceived as providers of species potent to degrade Tc.

**Figure 7:**
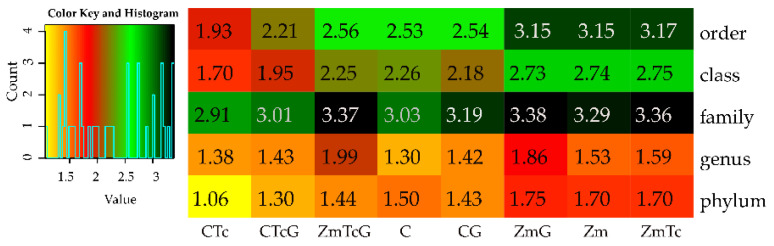
Diversity of bacterial taxa calculated from OTU according to Shannon-Wiener. C—non-sown soil (control), CTc—non-sown soil contaminated with tetracycline, CG—non-sown soil fertilized with compost, CTcG—non-sown soil contaminated with tetracycline and fertilized with compost, Zm—soil sown with maize (*Zea mays*), ZmTc—soil sown with maize and contaminated with tetracycline, ZmG—soil sown with maize and fertilized with compost, and ZmTcG—soil sown with maize, contaminated with tetracycline, and fertilized with compost.

Considering the above findings, it may be concluded that the changes observed in the genetic diversity of bacteria were reflected in the values of the Shannon-Wiener index (Figure 7). The contamination of non-sown soil with Tc diminished the diversity of bacteria at the phylum, class, order, and family levels. Soil fertilization with G decreased the diversity at the phylum and class levels, and increased that at the genus level. This increased bacterial diversity at the genus level was also observed in the soil not contaminated with Tc but fertilized with G. The greatest positive impact on bacterial diversity, found at all taxonomic levels, was elicited by Zm cultivation. In this experimental variant, Tc did not diminish the bacterial diversity. Soil fertilization with compost had little effect on the bacterial diversity, increasing it only at the genus level. Fertilization of the soil contaminated with Tc, likewise of the soil non-sown with Zm, diminished the bacterial diversity at the phylum, class, and order levels.

### 3.3. Response of Oxidoreductases to Soil Contamination with Tetracycline

Tetracycline was found to elicit a less pronounced effect on oxidoreductases than on soil bacteria (Table S2). IF_Tc_ of catalase was low (Table 7), ranging from −0.089 to 0.051, which indicates that Tc inhibited its activity by barely 8.9% or stimulated it by 5.1%. In turn, the IF_Tc_ values computed for dehydrogenases were always negative (ranging from −0.004 to −0.245), which proves a tendency for the inhibiting effect of Tc on these enzymes. The lowest IF_Tc_ values were noted for dehydrogenases on day 50 of the experiment in the soil sown with *Zea mays*. They reached −0.245 in the non-fertilized soil and −0.170 in the soil fertilized with compost.

The values of the IF_G_ index (Table 8) prove that fertilization with G promoted activities of oxidoreductases to a various extent depending on the analytical term, soil contamination with Tc, and cultivation of *Zea mays*. The strongest dehydrogenase stimulation was noted on day 50 of the experiment in the soil sown with Zm and contaminated with Tc (IF_G_ 0.742) and in the soil not contaminated with this antibiotic (IF_G_ 0.583), whereas the poorest one was in the non-sown soil (IF_G_ 0.022—0.046). The catalase activity was most strongly promoted by G fertilization in the non-sown soil not contaminated with Tc on day 50 of the experiment (IF_G_ 0.156) and in the Tc-contaminated soil on day 25 regardless of its cultivation (IF_G_ 0.141).

The cultivation of *Zea mays* elicited a significantly positive effect on the activity of dehydrogenases (Table 9), with a stronger stimulating effect observed on day 50 than 25 of the experiment. This effect was, however, significantly suppressed by Tc, as the IF_Zm_ value determined on day 50 of the experiment reached 0.982 in the non-contaminated soil not fertilized with G and 2.000 in the fertilized soil. Soil contamination with Tc decreased the IF_Zm_ value to 0.558 and 1.654, respectively. In contrast to dehydrogenases, the activity of catalase was modified by Zm cultivation to a little extent. In the case of this enzyme, apart from the null IF_Zm_ values noted on day 25 of the experiment in the soil contaminated with Tc, the maximal IF_Zm_ values were noted on day 50; however they were relatively low and ranged from 0.050 to 0.082.

## 4. Discussion

### 4.1. Effect of Tetracycline and Soil Fertilization on Plants

The continuous release of tetracyclines to the natural environment makes them ubiquitous in soil [36]. The growing concentrations of tetracycline group antibiotics in arable fields worldwide are especially alarming due to their potential impact on crops [70,71,72]. Antibiotics may exert direct phytotoxic effects contributing to, e.g., a decreased rate of seed germination [71], plant growth and biomass growth [73,74] as well as a decreased rate of respiration or chlorophyll synthesis [70]. In turn, in our study, Tc not only did not decrease the SPAD value but increased it at the 8th leaf stage. The effect of antibiotics is considerably stronger in hydroponic cultures than in soil. According to Liu et al. [71], in cultures of this type, the EC50 reached 57 mg dm^−3^ for oats, 69 mg dm^−3^ for rice, and as much as 203 mg dm^−3^ for cucumber. In turn, in soil cultures, the EC50 determined for these plants exceeded 300 mg kg^−1^ DM soil. This Tc dose analyzed in Phytotoxkits tests reduced the length of *Sinapis alba* L. roots by 40% [75]. The direct phytotoxic effect of antibiotics on plant growth can, undoubtedly, be estimated using phytotoxic kits [70,71,75], but determination of Tc impact on plants in pot and field experiments seems to be crucial. Our vegetation experiment demonstrated that Tc applied in a dose of 100 mg kg^−1^ soil did not inhibit the growth of both aerial parts and roots of *Zea mays*. This is consistent with the findings from the study by Chen et al. [76], where oxytetracycline doses of 15 and 200 mg kg^−1^ had no significant effect on the cultivation of *Amarantus mangestnus* L. and *Trifolium repens* L. The weaker inhibiting effect of tetracycline in the soil than in water may be attributed to its strong absorption in soil [36,58,77].

### 4.2. Effect of Tetracycline, Fertilization with Compost, and Cultivation of Zea Mays on Soil Bacteria Community

The release of increasing amounts of antibiotics to soil poses potential threats to all microorganisms colonizing this environment [18,24,78]. They can modify the structure and activity of soil bacteria communities [17]. The present study results demonstrated that soil contamination with Tc exerted selective pressure on soil microorganisms. The impact of antibiotics on soil microorganisms is strongly dependent on the extent of soil contamination [79,80]. The antibiotic tested in the present study (100 mg Tc kg^−1^ soil) inhibited the proliferation of organotrophic bacteria and actinobacteria, and promoted that of copiotrophic bacteria. Despite these changes, Tc had little effect upon the colony development index (CD) and ecophysiological diversity index (EP) of organotrophic bacteria and actinobacteria, probably due to its very high affinity to soil components. The percentage of Tc adsorption approximates 100%, whereas its desorption percentage falls under 10% [53].

Antibiotics, i.e., cyprofloxacin, oxytetracycline, sulfamethoxazol, and tylosin, applied in a dose of 1 mg kg^−1^ soil during cabbage, endive, and spinach cultivation had no significant effect on the population numbers of microorganisms [77]. In turn, Santás-Miguel et al. [79] demonstrated that soil contamination with a Tc dose of 2000 mg kg ^−1^ DM soil induced tolerance of the bacterial community to this antibiotic, which however diminished with time. A temporary effect of Tc applied in doses of 100 and 500 mg per 1 kg of soil was also demonstrated by Chessa et al. [58,81]. Its adverse effect on the activity and structure of microorganisms was observed to decline after 7 days and to disappear within 60 days. In the present study the effect of Tc on soil bacteriobiome was also observed to vary in time. On day 50 of the experiment, it was completely neutralized in the case of oligotrophic bacteria.

Antibiotics may modify the structure and composition of bacterial communities [74,78,82]. In the current study, Tc presence in the soil had a significant but varying effect on the abundance, diversity, and structure of bacterial communities colonizing it. At the phylum level, the bacterial community was mainly constituted by *Proteobacteria* and *Actinobacteria*. The increased OTU numbers of bacteria from the following genera: *Cellulosimicrobium*, *Nocardioides*, *Candidatus Solibacter*, *Streptomyces*, *Terracoccus*, *Arthrobacter*, *Phycicoccus*, *Kutzneria*, and *Amycolatopsis* belonging to the phylum *Actinobacteria* and bacteria from the genera: *Rhodoplanes*, *Burkholderia*, and *Rhodanobacter* belonging to the phylum *Proteobacteria* in the soils contaminated with Tc can be associated with a growing ability of these microorganisms to degrade this antibiotic [54,83] and use it as a source of carbon and energy [24,58]. Potentially, the adverse Tc effect on the bacteria from the phylum *Proteobacteria* represented by *Kaistobacter*, *Sphingomonas*, *Sphingobium*, *Luteibacter*, and *Steroidobacter* genera could have been masked by enhanced activities of other microorganisms capable of developing in the presence of Tc, probably, by using compounds released from lysed cells of microorganisms [58,79,84,85].

Chessa et al. [58] and Chen et al. [52] point to the fact that tetracyclines are antibiotic residues most often reported in manure and sewage sludge. Increased concentrations of antibiotics in soil cause the number of antibiotic-resistant bacteria to increase, which modifies the sensitivity of entire populations of microorganisms to the antibiotic [86,87] and increases the number of genes resistant to tetracyclines in the soil [88,89]. The prevailing bacterial phyla in the soil fertilized with manure containing 171.07–660.20 μg Tc kg^1^ were *Proteobacteria*, *Acidobacteria*, *Actinobacteria*, *Chloroflexi*, and *Bacteroidetes*, accounting for 85.2–92.4% of the total soil bacteria population [80]. The long-term use of sewage sludge and hen droppings increased the counts of *Proteobacteria*, *Acidobacteria*, *Actinobacteria*, and *Chloroflexi.* In addition, five bacterial phyla (*Chloroflexi*, *Planctomycetes*, *Firmicutes*, *Gemmatimonadetes*, and *Bacteroidetes*) were significantly correlated with antibiotic-resistance genes (ARG) in the soil [88]. The changes observed in the present study in the diversity of microbial communities upon the influence of Tc may also be reflected in the modified functions of soil microorganisms, which probably translates into soil metabolism. The present study results highlighted that, regardless of stress induced by soil contamination with Tc, fertilization with compost, and sowing the soil with *Zea mays*, the stability of the soil microbiome was very well described by the core microbiome being common for all soil types tested and represented by *Kaistobacter*, *Cellulosimicrobium*, and *Rhodoplanes*. In the non-sown soil contaminated with Tc, the core microbiome comprised *Kutzneria*, whereas in the soil sown with *Zea mays*—it was represented by *Ralstonia* and DA101. This is very precious information considering the paucity of data related to basic changes in the community of microorganisms involved in accelerated removal of tetracyclines. These microorganisms can be used for bioaugmentation of antibiotic-contaminated areas.

Soil supplementation with grass compost, which provides an easily available pool of organic compounds, has turned out to be of key importance during ecosystem adaptation to adverse environmental conditions caused by soil contamination with tetracycline. Compost was found to significantly stimulate the development of autochthonous microorganisms and alleviate the adverse effect of Tc on bacteria proliferation. Also other bioadsorbents seem to be a fine alternative for minimizing the adverse effect of tetracycline on soil microbiomes due to their low cost and their ability to adsorb toxic substances [23,53]. These include: pine bark and crushed clam shell [17], manure [23], and biocharcoal [90]. Definitely lower negative IF_Tc_ values obtained in the soil fertilized using G than in the non-fertilized soil prove that organic matter provided to the soil with compost mitigates the adverse effects of tetracycline on the soil microbiome. The simultaneous use of Tc and compost caused lesser changes in the bacterial phylum structure. Also other studies corroborated the usability of organic matter in restoring the stability of Tc-contaminated soil. Bovine manure modified the bacterial structure in the soil, enhanced microbiological activity, and contributed to the restoration of the microbiological structure in the soil with Tc addition [58]. According to Yue et al. [90], biocharcoal from manure accelerated the removal of tetracyclines and promoted the growth of bacteria potentially degrading tetracyclines (*Acidothermus*, *Sphingomonas*, and *Blastococcus),* which may be used for in situ remediation of tetracycline-contaminated soils. The above considerations allow for the conclusion that the use of compost, likewise the use of manure or biocharcoal, is advisable as—being the source of nutrients and microorganisms—it may accelerate microbiological degradation of tetracycline. In addition, due to its structure, compost may potentially improve the physical properties of soil by modifying its pH, water capacity, and structure. The usability of compost in accelerating the microbiological degradation of tetracycline was also confirmed by the Shannon index values. They prove that compost mitigated the adverse Tc effect on bacterial diversity at all taxonomic levels in the non-sown soil. In the soil sown with *Zea mays*, its alleviating impact was confirmed only at the genus level.

### 4.3. Effect of Tetracycline, Fertilization with Compost, and Cultivation of Zea Mays on Activities of Soil Oxidoreductases

Both the microorganisms and enzymes they produce are very good indicators of soil health [59,91,92,93,94], because they are sensitive to various changes proceeding in the environment [34,59,61]. The present study results demonstrate that activity of oxidoreductases depended on all variables tested, i.e., soil contamination with tetracycline, soil fertilization with compost, soil sowing with *Zea mays*, and experiment duration. The sensitivity of dehydrogenases and catalase to tetracycline was relatively low. This is consistent with sparse and inexplicit reports related to the effect of this group of antibiotics on the activity of soil enzymes [85,95]. Wei et al. [72] and Kessler et al. [95] emphasized that soil contamination with Tc impaired the enzymatic activity of soil, particularly in the case of dehydrogenases. According to Chen et al. [76], oxytetracycline doses of 15 and 200 mg kg^−1^ adversely affected activity of dehydrogenases, whereas cultivation of *Amarantus mangestnus* L. and Trifolium repens L. played an insignificant role in alleviating the adverse effect of this antibiotic. In turn, Liu [96] demonstrated a temporary enhancement of dehydrogenase activity in the soil contaminated with chlorotetracycline. In the present study, Tc only slightly enhanced catalase activity on day 50 of the experiment. Generally, dehydrogenases were more responsive to Tc compared to catalase, probably due to growth inhibition or death of sensitive microorganisms [18,83,97]. In turn, the stimulating effect of Tc on catalase activity, observed in the present study, might have been caused by enhanced proliferation of copiotrophic bacteria in the Tc-contaminated soils. Probably, these bacteria are capable of surviving in the presence of antibiotics, using them as sources of carbon [29].

The enhanced activities of dehydrogenases and catalase upon grass compost application results most likely from the response of organotrophic bacteria, copiotrophic bacteria, and actinobacteria to the nutrient loads supplied to their communities. Compost application to soil provides microorganisms with easily available substrates, e.g., carbohydrates [98,99,100]. This is indicative of a feedback between bacterial community and activity of oxidoreductases, because these are the microorganisms that trigger the enhancement in the activity of dehydrogenases [100,101]. The latter directly reflects the activity of soil microorganisms [102,103] because dehydrogenases are endogenous enzymes responsible for bio-oxidation of soil organic matter [104,105].

The results of our study demonstrate that tetracycline disturbed the stability of a soil ecosystem and confirm the hypothesis that *Zea mays* cultivation and soil fertilization with good-quality compost are utile in restoring the biological homeostasis of soil contaminated with this antibiotic. This finding suggests the permanent need for searching, developing, and implementing strategies for the bioremediation of soils contaminated with antibiotics.

## 5. Conclusions

Tetracycline (Tc) present in the soil in a dose of 100 mg kg^−1^ did not inhibit the yield of *Zea mays* (Zm) and did not decrease its leaf greenness index (SPAD). Even though it impaired the proliferation of culture bacteria, it did not affect the values of the colony development index (CD) and the ecophysiological diversity index (EP). Tetracycline exerted a positive effect on copiotrophic bacteria and an adverse effect on organotrophic bacteria, actinobacteria, oligotrophic bacteria as well as soil oxidoreductases. The bacteria belonging to *Acidiobacteria* and *Proteobacteria* phyla were found to be prevailing soil bacteria. In the non-sown soil, tetracycline increased the relative abundance of *Acidiobacteria* by 19% and reduced that of *Proteobacteria* by 9%. In the soil sown with Zm, the Tc effect on the relative abundance of all bacterial phyla was minor and changes observed ranged from −1.29% to 1.25%, which was mainly due to the significant positive impact of Zm on bacteria of the identified phyla. The greatest changes in the structure of *Actinobacteria* and *Proteobacteria* were caused by Zm cultivation, as it decreased the relative OTU number of *Actinobacteria* by 21% and increased that of *Proteobacteria* by 14%. Fertilization with grass compost (G) and Zm cultivation alleviated its adverse effect on the mentioned groups of bacteria and activities of soil dehydrogenases and catalase. The metagenomic analysis demonstrated that Tc application and fertilization of non-sown soil with G as well as Zm cultivation significantly increased the OTU numbers of bacteria. Soil fertilization with G reduced the relative abundance of *Acidiobacteria* and *Actinobacteria* as well as increased *Proteobacteria* abundance in both soil variants. The *Kaistobacter* and *Rhodoplanes* bacteria belonging to the phylum *Proteobacteria* and *Cellulosimicrobium* belonging to the phylum *Actinobacterium* constituted the core microbiome in both the soil sown with Zm and in the non-sown soil. In turn, specific genera identified exclusively in the Tc-contaminated soil turned out to be *Kutzneria (p_Actinobacteria), DA101 (p_Verrumicrobe)*, and *Ralstonia (p_Actinobacteria).* The mentioned genera should be perceived as sources of species effective in bioaugmentation of soils contaminated with Tc. In the non-sown soil, Tc diminished diversity of bacteria at all taxonomic levels, except for the genus level. This unfavorable phenomenon was mitigated by soil fertilization with compost and by *Zea mays* cultivation.

The present study recommends soil fertilization with grass compost and *Zea mays* cultivation in bioremediation of soils contaminated with tetracycline and indicates the usability of the so-called core bacteria, developing well in the presence of tetracycline, for the bioaugmentation of soils contaminated with this antibiotic.

## Figures and Tables

**Table 1:** The concentrations of tetracycline in soil reported in other countries and regions.

Location	Soil Sample Description	Maximum Antibiotics, Concentration, µg kg^−1^	References
city of Shenyan, northeast China	depth of 0–15 cm	976.17	[20]
China, Beijing,	soils from vegetable greenhouses	74.4	[21]
Spain, South surrounding of Valencia	agricultural soils with 20% of clay	64.4	[22]
Korea, Hongcheon, Gangwon province,	paddy and upland soils	177.6	[19]
Spain, NW, Galicia	sand 40%, silt—38%, clay—22%	600	[23]
Germany	0–10, 10–20 and 20–30 cm soils sand 91.6%, silt—6.0%, clay—2.4%	86.2; 198.7; 171.7	[24]
Poland, areas of Pomeranian Voivodeship	soils from agricultural areas of Pomeranian Voivodeship	14.5	[25]

**Table 2:** Some properties of the soil and compost used in the experiment, the test units and literature according to which the analyses were made.

Abbreviation	Properties	Unit	Soil	Compost	References
Chemical and physicochemical properties					
N_tot_	total nitrogen	g kg^−1^ DM	0.83 ± 0.05	20.18 ± 0.86	[39]
C_org_	organic carbon		10.00 ± 0.60	146.61 ± 6.19	[40]
SOM	soil organic matter		17.24 ± 1.03	252.76 ± 10.67	[40]
P	phosphorus	mg kg^−1^ DM	81.10 ± 4.13	3.41 ± 0.18	[41]
K	potassium		145.25 ± 7.18	9.25 ± 0.24	[41]
Mg	magnesium		71.00 ± 3.00	5.69 ± 0.17	[42]
pH	pH_KCl—_soil reaction	1 mol KCl dm^−3^	4.40 ± 0.15	6.1 ± 0.15	[43]
EBC	sum of exchangeable base cations	mmol (+) kg^−1^ DM	63.60 ± 4.50	659.76 ± 32.01	[44]
HAC	hydrolytic acidity		26.10 ± 1.92	82.04 ± 3.98	[44]
CEC	cation exchange capacity		89.70	741.80	[44]
ACS	alkaline cation saturation	%	70.90	88.94	[44]
Exchangeable cations					
K^+^	potassium	mg kg^−1^ DM	168.00 ± 8.30	NA	[45]
Ca^++^	calcium		1190.5 ± 52.70	NA	[45]
Na^+^	sodium		10.00 ± 0.43	NA	[45]
Mg^++^	magnesium		82.10 ± 4.70	NA	[45]
Microorganisms number per 1 kg DM					
Org	organotrophic bacteria	cfu	36.728 ± 1.986 × 10^9^	NA	[46]
Olig	oligotrophic bacteria		7.740 ± 0.581 × 10^9^	NA	[47]
Cop	copiotrophic bacteria		11.240 ± 0.127 × 10^9^	NA	[47]
Act	actinomyces		17.008 ± 0.537 × 10^9^	NA	[48]
Enzymatic activity per 1 kg DM h^−1^					
Deh	dehydrogenases	µmol TFF	4.042 ± 0.136	NA	[49]
Cat	catalase	mol O_2_	0.212 ± 0.001	NA	[50]

NA—not analized.

**Table 3:** The yield of *Zea mays* and the value of leaf greenness index (SPAD).

Tc Content (mg kg^−1^ DM of soil)	Yield, (DM g pot^−1^)			*Zea Mays* Development Phase SPAD	
	Shoots	Roots	Together	4 Leaves	8 Leaves
−G					
0	55.33 *^b^* ± 0.92	13.51 *^bc^* ± 0.44	68.84 *^c^* ± 0.44	38.10 *^a^* ± 2.74	22.62 *^b^* ± 4.89
100	56.49 *^b^* ± 1.55	14.69 *^ab^* ± 0.42	71.18 *^bc^* ± 0.64	38.69 *^a^* ± 3.62	28.00 *^a^* ± 2.36
+G					
0	60.46 *^a^* ± 0.93	12.07 *^c^* ± 1.44	72.53 *^b^* ± 1.12	34.36 *^c^* ± 2.36	29.01 *^a^* ± 1.54
100	62.48 *^a^* ± 1.03	15.83 *^a^* ± 0.72	78.31 *^a^* ± 1.37	36.33 *^b^* ± 2.61	27.48 *^a^* ± 1.58

Tc—tetracycline; −G—soil without grass compost; +G—soil with grass compost. Homogeneous groups denoted with letters (*a*–*c*) were calculated separately for each of the columns.

**Table 4:** The index of tetracycline effect on the number of microorganisms (IF_Tc_).

The Dose of Grass Compost in g C kg^−1^ DM Soil	−Zm		+Zm	
	Analysis Day			
	25	50	25	50
Organotrophic bacteria (Org)				
0	−0.352 *^d^* ± 0.011	−0.514 *^e^* ± 0.046	−0.122 *^ab^* ± 0.007	−0.123 *^ab^* ± 0.014
4	−0.093 *^ab^* ± 0.005	−0.161 *^bc^* ± 0.012	−0.236 *^c^* ± 0.025	−0.061 *^a^* ± 0.002
Oligotrophic bacteria (Olig)				
0	−0.262 *^f^* ± 0.022	−0.009 *^d^* ± 0.001	−0.469 *^g^* ± 0.027	−0.052 *^de^* ± 0.005
4	−0.102 *^e^* ± 0.008	0.223 *^b^* ± 0.017	0.687 *^a^* ± 0.029	0.134 *^c^* ± 0.008
Copiotrophic bacteria (Cop)				
0	0.214 *^c^*^–*e*^ ± 0.015	0.375 *^bc^* ± 0.053	0.300 *^cd^* ± 0.064	0.591 *^a^* ± 0.007
4	0.070 *^e^* ± 0.006	0.388 *^bc^* ± 0.051	0.139 *^de^* ± 0.005	0.529 *^ab^* ± 0.051
Actinomycetes (Act)				
0	−0.633 *^c^* ± 0.060	−0.265 *^b^* ± 0.067	−0.063 *^b^* ± 0.006	−0.259 *^b^* ± 0.016
4	−0.222 *^ab^* ± 0.021	−0.217 *^ab^* ± 0.037	−0.054 *^a^* ± 0.005	−0.118 *^ab^* ± 0.53

−Zm—unsown soil; +Zm—soil sown with *Zea mays.* Homogeneous groups denoted with letters (*a*–*g*) were calculated separately for each group of microorganisms.

**Table 5:** The index of compost effect on the number of microorganisms (IF_G_).

Tc Content (mg kg^−1^ DM of soil)	−Zm		+Zm	
	Analysis Day			
	25	50	25	50
Organotrophic bacteria (Org)				
0	0.289 *^c^* ± 0.060	0.150 *^c^*^–*e*^ ± 0.015	0.203 *^cd^* ± 0.021	−0.060 *^f^* ± 0.014
100	0.804 *^b^* ± 0.083	0.988 *^a^* ± 0.047	0.046 *^d^*^–*f*^ ± 0.004	0.005 *^ef^* ± 0.002
Oligotrophic bacteria (Olig)				
0	−0.333 *^e^* ± 0.029	−0.118 *^cd^* ± 0.019	−0.353 *^e^* ± 0.045	−0.189 *^d^* ± 0.017
100	−0.189 *^d^* ± 0.007	0.089 *^b^* ± 0.023	1.057 *^a^* ± 0.036	−0.031 *^c^* ± 0.010
Copiotrophic bacteria (Cop)				
0	0.190 *^ab^* ± 0.019	0.058 *^c^* ± 0.020	0.278 *^a^* ± 0.033	0.074 *^c^* ± 0.036
100	0.049 *^c^* ± 0.005	0.068 *^c^* ± 0.015	0.120 *^ab^* ± 0.053	0.032 *^c^* ± 0.021
Actinomycetes (Act)				
0	1.020 *^b^* ± 0.075	0.878 *^bc^* ± 0.034	0.311 *^d^* ± 0.029	0.528 *^cd^* ± 0.078
100	3.278 *^a^* ± 0.374	1.000 *^b^* ± 0.146	0.323 *^d^* ± 0.043	0.819 *^bc^* ± 0.089

Tc—tetracycline; −Zm—unsown soil; +Zm—soil sown with *Zea mays*. Homogeneous groups denoted with letters (*a*–*f*) were calculated separately for each group of microorganisms.

**Table 6:** The index of *Zea mays* effect on the number of microorganisms (IF_Zm_).

Tc Content (mg kg^−1^ DM of Soil)	Microorganisms							
	Org		Olig		Cop		Act	
	Analysis Day							
	25	50	25	50	25	50	25	50
−G								
0	0.563 *^e^* ± 0.049	1.405 *^b^* ± 0.049	0.417 *^b^* ± 0.008	0.402 *^b^* ± 0.019	0.071 *^bc^* ± 0.007	0.027 *^c^* ± 0.009	1.429 *^b^* ± 0.114	1.204 *^ab^* ± 0.066
100	1.120 *^c^* ± 0.016	3.345 *^a^* ± 0.069	0.018 *^d^* ± 0.005	0.342 *^ab^* ± 0.027	0.147 *^a^*^–*c*^ ± 0.057	0.188 *^ab^* ± 0.022	5.194 *^a^* ± 0.547	1.222 *^ab^* ± 0.049
+G								
0	0.459 *^f^* ± 0.025	0.965 *^d^* ± 0.029	0.375 *^ab^* ± 0.038	0.289 *^ab^* ± 0.039	0.150 *^a^*^–*c*^ ± 0.048	0.042 *^c^* ± 0.012	0.576 *^c^* ± 0.095	0.793 *^ab^* ± 0.093
100	0.229 *^g^* ± 0.039	1.198 *^c^* ± 0.015	1.583 *^a^* ± 0.045	0.194 *^cd^* ± 0.018	0.224 *^a^* ± 0.028	0.148 *^a^*^–^*^c^* ± 0.038	0.916 *^ab^* ± 0.098	1.021 *^ab^* ± 0.101

Tc—tetracycline; −G—soil without grass compost; +G—soil with grass compost. Org—organotrophic bacteria, Olig—oligotrophic bacteria, Cop—copiotrophic bacteria, Act—actinomycetes. Homogeneous groups denoted with letters (*a*–*g*) were calculated separately for each group of microorganisms.

**Table 7:** The index of tetracycline effect on the activity of soil enzymes (IF_Tc_).

The Dose of Grass Compost in g C kg^−1^ DM Soil	−Zm		+Zm	
	Analysis Day			
	25	50	25	50
Dehydrogenases				
0	−0.087 *^b^* ± 0.016	−0.040 *^ab^* ± 0.008	−0.074 *^b^* ± 0.018	−0.245 *^d^* ± 0.021
4	−0.004 *^a^* ± 0.001	−0.061 *^ab^* ± 0.009	−0.078 *^b^* ± 0.016	−0.170 *^c^* ± 0.013
Catalase				
0	−0.042 *^b^* ± 0.010	0.047 *^a^* ± 0.010	−0.089 *^c^* ± 0.014	0.051 *^a^* ± 0.002
4	0.016 *^a^* ± 0.004	−0.054 *^bc^* ± 0.012	−0.037 *^b^* ± 0.010	−0.026 *^b^* ± 0.004

−Zm—unsown soil; +Zm—soil sown with *Zea mays.* Homogeneous groups denoted with letters (*a*–*d*) were calculated separately for each enzyme.

**Table 8:** The index of compost effect on the activity of soil enzymes (IF_G_).

Tc Content (mg kg^−1^ DM of Soil)	−Zm		+Zm	
	Analysis Day			
	25	50	25	50
Dehydrogenases				
0	0.061 *^d^* ± 0.053	0.046 *^d^* ± 0.005	0.043 *^d^* ± 0.011	0.583 *^b^* ± 0.014
100	0.158 *^c^* ± 0.008	0.022 *^d^* ± 0.033	0.038 *^d^* ± 0.018	0.742 *^a^* ± 0.028
Catalase				
0	0.076 *^bc^* ± 0.005	0.156 *^a^* ± 0.005	0.081 *^bc^* ± 0.028	0.094 *^b^* ± 0.005
100	0.141 *^a^* ± 0.015	0.045 *^cd^* ± 0.013	0.141 *^a^* ± 0.015	0.014 *^d^* ± 0.012

Tc—tetracycline; −Zm—unsown soil; +Zm—soil sown with *Zea mays.* Homogeneous groups denoted with letters (*a*–*d*) were calculated separately for each enzyme.

**Table 9:** The index of *Zea mays* effect on the activity of soil enzymes (IF_Zm_).

Tc Content (mg kg^−1^ DM of Soil)	Dehydrogenases		Catalase	
	Analysis Day			
	25	50	25	50
−G				
0	0.237 *^ef^* ± 0.007	0.982 *^c^* ± 0.055	0.051 *^ab^* ± 0.003	0.078 *^a^* ± 0.014
100	0.254 *^e^* ± 0.025	0.558 *^d^* ± 0.040	0.000 *^c^* ± 0.003	0.082 *^a^* ± 0.003
+G				
0	0.215 *^ef^* ± 0.012	2.000 *^a^* ± 0.026	0.055 *^ab^* ± 0.007	0.020 *^bc^* ± 0.003
100	0.124 *^f^* ± 0.019	1.654 *^b^* ± 0.043	0.000 *^c^* ± 0.003	0.050 *^ab^* ± 0.005

Tc—tetracycline; −G—soil without grass compost; +G—soil with grass compost. Homogeneous groups denoted with letters (*a*–*f*) were calculated separately for each enzyme.

## Data Availability

Sequencing data has been deposited with GenBank NCBI. They are available online: https://www.ncbi.nlm.nih.gov/nuccore/?term=ON042235:ON042332[accn] (accessed on 27 March 2022) under accession numbers ON042235–ON042332.

## Supplementary Materials

The following supporting information can be downloaded at: https://www.mdpi.com/article/10.3390/ijerph19127357/s1, Table S1. The number of bacteria, 10^9^ cfu kg^−1^ DM of soil; Table S2: Enzymatic activity in soil, kg^−1^ DM of soil h^−1^.
