# Supplementary material for: The Role of Grass Compost and Zea Mays in Alleviating Toxic Effects of Tetracycline on the Soil Bacteria Community
Source: Int J Environ Res Public Health. 2022 Jun 15;19(12):7357. doi: 10.3390/ijerph19127357 (PMC9223702; doi:10.3390/ijerph19127357)
Supplement: Supplementary file 1 [file ijerph-19-07357-s001.zip › ijerph-1731531-supplementary.pdf]

International Journal of
Environmental Research and Public Health

MDPI

Supplementary Materials

The Role of Grass Compost and *Zea mays* in Alleviating Toxic Effects of Tetracycline on the Soil Bacteria Community

Jadwiga Wyszkowska*, Agata Borowik and Jan Kucharski

Department of Soil Science and Microbiology, University of Warmia and Mazury in Olsztyn, 10-727 Olsztyn, Poland; agata.borowik@uwm.edu.pl (A.B.); jan.kucharski@uwm.edu.pl (J.K.)

* Correspondence: jadwiga.wyszkowska@uwm.edu.pl (J.W.)

Table S1. The number of bacteria, 10^9 cfu kg^{-1} DM of soil.

Tc content (mg kg^{-1} DM of soil)	-Zm		+Zm	
	Analysis day			
	25	50	25	50
		Organotrophic bacteria (Org)		
		-G		
0	7.001^{i}	8.529^{h}	10.945^{e}	20.509^{a}
100	4.536^{j}	4.141^{j}	9.613^{fg}	17.994^{c}
		+G		
0	9.022^{gh}	9.811^{fg}	13.163^{d}	19.276^{b}
100	8.184^{h}	8.233^{h}	10.057^{ef}	18.093^{c}
		Oligotrophic bacteria (Olig)		
		-G		
0	25.439^{c}	17.312^{ef}	36.038^{b}	24.273^{c}
100	18.783^{e}	17.156^{ef}	19.128^{e}	23.023^{c}
		+G		
0	16.959^{ef}	15.275^{f}	23.319^{c}	19.687^{de}
100	15.234^{f}	18.685^{e}	39.341^{a}	22.315^{cd}
		Copiotrophic bacteria (Cop)		
		-G		
0	4.141^{h}	4.768 $^{f-g}$	4.437^{gh}	4.896 $^{e-h}$
100	5.029 $^{e-g}$	6.557^{cd}	5.768^{de}	7.789^{ab}
		+G		
0	4.930 $^{e-h}$	5.045^{e-h}	5.669^{d-f}	5.257^{e-g}
100	5.275^{e-g}	7.001^{bc}	6.458^{cd}	8.040^{a}
		Actinomycetes (Act)		
		-G		
0	4.831^{f}	4.831^{f}	11.733^{b}	10.649^{bc}
100	1.775^{g}	3.550^{fg}	10.994^{bc}	7.888^{de}
		+G		
0	9.761^{b-d}	9.071^{c-e}	15.382^{a}	16.269^{a}
100	7.59^{2de}	7.099^{e}	14.543^{a}	14.346^{a}

Tc – tetracycline; -G - soil without grass compost; +G - soil with grass compost; -Zm – unsown soil; +Zm - soil sown with *Zea mays*. Homogeneous groups denoted with letters (a–j) were calculated separately for each group of microorganisms.

Table S2. Enzymatic activity in soil, kg^{-1} DM of soil h^{-1}.

Tc content (mg kg^{-1} DM of soil)	-Zm		+Zm	
	Analysis day			
	25	50	25	50
Dehydrogenases, μmol TFF				
-G				
0	4.899^{d}	1.601^{h}	6.059^{ab}	3.172^{f}
100	4.473^{e}	1.537^{h}	5.608^{c}	2.394^{g}
+G				
0	5.200^{d}	1.674^{h}	6.317^{a}	5.023^{d}
100	5.178^{d}	1.571^{h}	5.823^{bc}	4.171^{e}
Catalase, mol O_2				
-G				
0	0.305^{j}	0.338^{gh}	0.321^{i}	0.365^{de}
100	0.292^{j}	0.354^{ef}	0.292^{j}	0.383^{bc}
+G				
0	0.328^{hi}	0.391^{ab}	0.347^{fg}	0.399^{a}
100	0.334^{g-i}	0.370^{cd}	0.334^{g-i}	0.389^{ab}

Tc – tetracycline; -G - soil without grass compost; +G - soil with grass compost; -Zm – unsown soil; +Zm - soil sown with *Zea mays*. Homogeneous groups denoted with letters (a–j) were calculated separately for each enzyme.
